# New Biotinylated GHK and Related Copper(II) Complex: Antioxidant and Antiglycant Properties In Vitro against Neurodegenerative Disorders

**DOI:** 10.3390/molecules28186724

**Published:** 2023-09-20

**Authors:** Rita Tosto, Graziella Vecchio, Francesco Bellia

**Affiliations:** 1 Institute of Crystallography, National Research Council of Italy (CNR), P. Gaifami 18, 95126 Catania, Italy; rita.tosto@ic.cnr.it; 2 Department of Chemical Sciences, University of Catania, A. Doria 6, 95125 Catania, Italy; gr.vecchio@unict.it

**Keywords:** peptide, Alzheimer, acrolein, copper, amyloid, antioxidant

## Abstract

Neurodegenerative diseases affect millions of people worldwide. The failure of the enzymatic degradation, the oxidative stress, the dyshomeostasis of metal ions, among many other biochemical events, might trigger the pathological route, but the onset of these pathologies is unknown. Multi-target and multifunctional molecules could address several biomolecular issues of the pathologies. The tripeptide GHK, a bioactive fragment of several proteins, and the related copper(II) complex have been largely used for many purposes, from cosmetic to therapeutic applications. GHK derivatives were synthesized to increase the peptide stability and improve the target delivery. Herein we report the synthesis of a new biotin–GHK conjugate (BioGHK) through orthogonal reactions. BioGHK is still capable of coordinating copper(II), as observed by spectroscopic and spectrometric measurements. The spectroscopic monitoring of the copper-induced ascorbate oxidation was used to measure the antioxidant activity Cu(II)-BioGHK complex, whereas antiglycant activity of the ligand towards harmful reactive species was investigated using MALDI-TOF. The affinity of BioGHK for streptavidin was evaluated using a spectrophotometric assay and compared to that of biotin. Finally, the antiaggregant activity towards amyloid-β was evaluated using a turn-on fluorescent dye. BioGHK could treat and/or prevent several adverse biochemical reactions that characterize neurodegenerative disorders, such as Alzheimer’s disease.

## 1. Introduction

Neurodegenerative diseases affect millions of people worldwide, and the spread of these devastating disorders is going to be even worse in the next decades [1]. They lack an effective therapy since the onset of these pathologies is unknown yet. However, it has been found that several common biochemical events might trigger the pathological route, such as the failure of the enzymatic degradation, the oxidative and nitrosative stress, the dyshomeostasis of metal ions, among many others [2]. Many approaches have been used to limit the progression of these disorders. A shared idea is using molecules that could exert many beneficial activities in vivo in order to address several biomolecular issues of the pathologies.

GHK (Gly-His-Lys) is a well-known matricryptin, i.e., a bioactive fragment released from the extracellular proteins by limited proteolysis having biological activities on its own [3]. GHK originates from several sources, such as type I collagen and SPARC protein [4], but it is also released from proteolytically damaged proteins during wound healing [5]. This naturally occurring tripeptide has been used for many purposes, mainly for wound healing and skin care [6]. Nonetheless, the number of applications has been increasing when the peptide is coordinated by copper(II). GHK-Cu(II) complex shows bioavailability, antioxidant and anti-inflammatory activities better than that reported for the tripeptide alone [7,8]. The regenerative and protective actions mainly for the skin tissues make GHK-Cu(II) largely used for cosmetic applications. However, the biological effects exerted by GHK and GHK-Cu(II) are relevant to cancer [9,10,11,12], neurodegeneration disorders and cognitive health [13,14,15].

The conjugation of GHK to several cofactors has been used as a strategy to (i) increase the stability of the tripeptide towards the protease action, (ii) enlarge the number of biological functions, and (iii) improve the delivery to specific targets [16]. Biotin was linked through the N-terminal amino group and the Lys side chain [17,18]. The derivatization of GHK with biotin was carried out in order to reach all these scopes. Indeed, biotinylated GHK, also incorporated into a collagenous matrix, could be used as a therapeutic agent in wound healing processes in vitro and in vivo [17,18], and in diabetic models [19].

**Figure 1:**
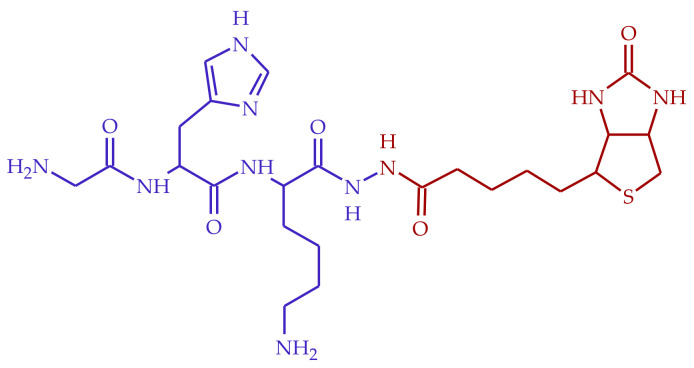
Structure of BioGHK, formed by the GHK (blue) and the biotin (red) moieties.

Herein we report the synthesis of a new biotin– GHK conjugate (BioGHK), in which the biotin cofactor is linked to the tripeptide through the C-terminal carboxylic group (Figure 1). Such a strategy preserves the copper(II)-binding ability of the GHK sequence, as proved by spectroscopic and spectrometric measurements. The biotin group makes BioGHK able to interact with streptavidin, a well-known approach for drug delivery [20]; it also preserves the properties of GHK as an antiglycating agent against the production of carbonylated species of amyloid-β (Aβ), a protein whose dyshomeostasis is involved in the onset/progression of Alzheimer’s disease (AD). Moreover, both GHK and BioGHK inhibit the ROS production catalyzed by the Cu(II)-Aβ complex system. Finally, BioGHK inhibits the amyloid-type aggregation of Aβ better than its parent compounds.

## 2. Results and Discussion

### 2.1. Synthesis and Purification of BioGHK

The reaction between GHK and biotinyl-hydrazide requires the protection of the two primary amino groups of the peptide, namely, the N-terminal and Lys side-chain amino groups. The protection reaction was carried out with the anhydride of t-butyloxy-pyrocarbonate (BOC). The chromatogram related to the purification of the reaction mixture (Figure S1A) shows the presence of two peaks (RT 14.5 and 23.1 min) due to reaction products, subsequently assigned, by ESI-MS analysis, to the GHK-(mono)-Boc and to the GHK-di-Boc conjugates. The ^1^H NMR spectra confirmed the identity of the compounds.

The second peak, eluted in the isocratic step at 100% of methanol, was collected. The conjugation of biotinyl-hydrazide to GHK-di-BOC was carried out using HOBt and DCC as the coupling agents in order to avoid racemization. The subsequent addition of biotinyl-hydrazide to the mixture gives rise to the reaction with GHK. After the deprotection of both the amino groups of glycine and lysine in TFA, the final product was purified by reverse-phase chromatography (Figure S1B). The unreacted compound, the other reagents and the soluble by-products elute in the first step of the purification process, whereas the main product elutes during the first part of H_2_O-CH_3_OH gradient, at 14.5 min. The identity of the final product was confirmed by ESI-MS and ^1^H NMR investigations.

### 2.2. NMR Characterization of BioGHK

**Figure 2:**
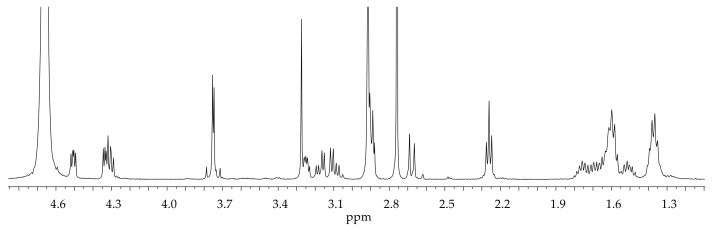
^1^H NMR spectrum of BioGHK (500 MHz, D_2_O).

The ^1^H NMR spectrum of BOC-protected GHK is reported in Figure S2, whereas Figure 2 shows the monodimensional NMR spectrum of BioGHK. The signals have been attributed using COSY (Figure S3A) and TOCSY (Figure S3B) spectra.

In the spectrum of the BOC-protected peptide (Figure S2), the peaks due to the imidazole protons are detectable (δ = 7.22 and 8.41 ppm); the group 18 protons include those of methyl groups belonging to the protecting groups, some of the lysine side chain (δ = 1.32 ppm–1.57 ppm), and the biotin valerate chain. In the range between 3 and 5 ppm, the α protons of the amino acid residues and the two diastereotopic protons of the histidine side chain resonate at 3.20 and 3.39 ppm, respectively.

The aromatic region (1.3–4.8 ppm) does not show any difference to that obtained for the GHK-di-BOC; in this range, it is possible to appreciate the main peaks related to biotin, as well as those of GHK shifted upon the reaction with biotin. In the high-field region, the alkyl groups of the valerate chain are close to the signals due to the side chain of lysine. Characteristic signals of biotin are: the triplet at 2.26 ppm due to the alpha protons to the hydrazide group and the doublet at 2.67 ppm due to one of the diastereotopic protons proximal to the sulfur ion. It is possible to identify the signals due to the protons of the heterocyclic rings in the same region in which the α amino acid protons resonate.

### 2.3. Structural Characterization of the Ligand

The new conjugate of GHK with biotin was structurally characterized using ESI Mass Spectrometry.

**Figure 3:**
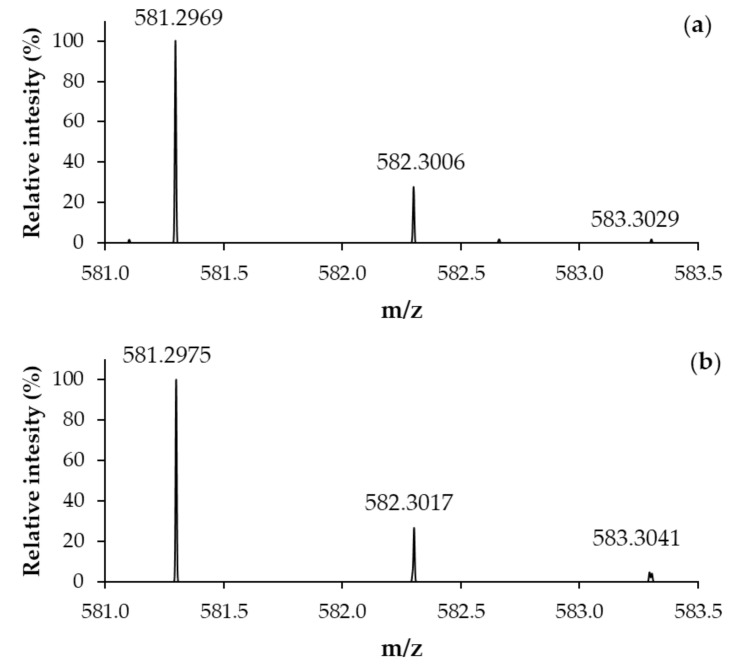
(**a**) Experimental and (**b**) simulated ESI-MS spectrum (Zoom scan) of BioGHK ([M + H]^+^).

The ESI-MS spectrum of BioGHK (Figure S4) clearly shows the presence of three main peaks at *m*/*z* 291.45, 581.42 and 1161.40. The base peak was assigned to the double-charged protonated species [M + 2H]^2+^, while the other ones are related to protonated mono-charged adducts ([M + H]^+^ and [2M + H]^+^, respectively). The identity of BioGHK has been assessed by comparing the experimental isotopic distribution of the mono-charged adduct ([M + H]^+^) to the simulated one (Figure 3).

The formation of single and double positively charged species is due to three basic groups (the imidazole nitrogen atoms, the N-terminal and the lysine side-chain amino groups) and a unique acidic group (the C-terminal carboxylate). The attribution of the species to the peaks reported in the MS spectrum was also possible through the Zoom Scan analysis (Figure S5). The isotopic distribution of the peak at *m*/*z* 291.5 (Figure S5A) is typically due to a double-charged species (the *m*/*z* differences between adjacent isotopic peaks is 0.5), whereas Zoom Scan spectra of the species at *m*/*z* 581.4 (Figure S5B) and 1161.4 (Figure S5C) are clearly ascribed to a mono-charged species. The other peaks in the ESI mass spectrum of BioGHK detected at *m*/*z* 683.4 and 1183.4 were assigned to mono-charged sodium adducts ([M + Na]^+^ and [2M + Na]^+^, respectively).

The structural sequence of BioGHK was confirmed by performing the multidimensional fragmentation (MS^n^). The spectra (MS^2^, MS^3^ and MS^4^) produced for the analysis of BioGHK are shown in Figure S6. Indeed, the selected mono-charged protonated adduct of BioGHK (*m*/*z* 581.3, Figure S6A) was fragmented to produce different signals (MS^2^@581.3, Figure S6B), whose highest intense peak was isolated (*m*/*z* 323.2, Figure S6C) and fragmented (MS^3^@323.2, Figure S6D). The last species investigated (*m*/*z* 305.8) was first isolated (Figure S6E) and then properly fragmented (MS4@ 305.8, Figure S6F).

All the species produced by multidimensional degradation form after the loss of neutral species from the isolated species and the detected signals are mainly due to the formation b- and y-type species. Figure S7 summarizes the b- and y-type series that BioGHK can form, whereas Table 1 shows the species detected in the MS/MS spectrum and the comparison to the calculated *m*/*z* values.

The MS/MS fragmentation of the mono-charged protonated adduct of BioGHK, (*m*/*z* 581.3) (Figure S7 and Table 1) produced all the b-type fragments, except for b1 whose *m*/*z* value (58.03) is outside the mass range of analysis (*m*/*z* 150–700). The fragmentation of the b3 ion (*m*/*z* 323.2, Figure S6D) mainly leads to the loss of a water molecule, whereas the subsequent fragmentation (Figure S6F) induces the removal of an ammonia molecule. The loss of small neutral molecules, such as H_2_O and NH_3_, is typical of the fragmentation of protonated peptide systems.

### 2.4. Interaction Sav/BioGHK

**Figure 4:**
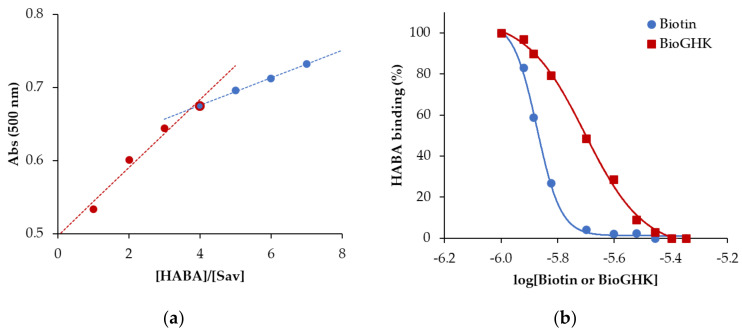
(**a**) Absorbance variation at 500 nm as a function of the [HABA]/[Sav] ratio. (**b**) Calculation of the IC50 for biotin (dashed line) and BioGHK (straight line).

The affinity of BioGHK for streptavidin was evaluated using a spectrophotometric assay and compared to that of biotin. For this purpose, we used 2-(4-hydroxyphenylazo)-benzoic acid (HABA), which increases the absorbance at 500 nm upon binding to streptavidin. The first part of the assay was used to determine the number of binding sites HABA saturates when interacting with streptavidin. The ratio between the absorbance of the substrate–protein complex and that of the ligand alone (Abs/Abs_0_) has been reported as a function of the ligand-to-protein ratio (Figure 4a). A clear discontinuity at the value of [HABA]/[Sav] equal to 4 confirms the number of binding sites of streptavidin and therefore represents a validation of the spectrophotometric assay. Then, we tested the interaction of the new GHK derivative with the Sav whose active sites were previously saturated with HABA. Since the affinity of HABA for Sav (Kd 5.8 × 10^−6^) is much lower than that of protein for biotin (Kd ≈ 10–15), when BioGHK or biotin is added to the mixture, HABA is substituted in the complex with Sav, and absorbance at 500 nm is reduced. This effect can be clearly seen in Figure S8, which shows the variations in the absorbance upon adding BioGHK. These spectrophotometric titrations were used to calculate the biotin concentration required to replace 50% of the sites occupied by HABA (IC50). This parameter is linked to the Hill index (nH), which measures the cooperative trend of the process. These data are obtained by plotting the absorbance values at 500 nm during titration as a function of the logarithm of the added biotin or BioGHK concentration (Figure 4b) and interpolating the data following Equation (1):

(1) $$F\left(x\right)=min+\frac{max-min}{1+10^{(log{IC}_{50}-x)n_{H}}}$$

where the minimum (*F*_0_) and maximum (*F_max_*) absorbance values have been normalized and expressed in terms of relative percentage with respect to the maximum absorbance value recorded.

The IC_50_ values of biotin alone (22.6 ± 0.9 µM) and conjugated to GHK (30.0 ± 2.7 µM) are not significantly different; moreover, the values of the Hill index for both the protein interactions with biotin (−0.11 ± 0.02) and the peptide derivative (−0.06 ± 0.03) are non-cooperative. These data confirm those reported in the literature [21], and BioGHK does not show drastic differences compared to the free vitamin in terms of interaction with Sav.

### 2.5. Characterization of the Cu-BioGHK System

Mass spectrometry has proved to be an excellent method for determining the stoichiometry of copper complexes with peptides [22,23,24,25]. For this reason, complex copper(II) species with BioGHK were characterized by ESI-MS. The MS spectra of the Cu^2+^/BioGHK system at a 1:1 metal-to-ligand molar ratio were recorded, exploring pH values from 5 to 8 (Figure S9).

The base peak in the MS spectrum of the Cu-GHK complex at pH 5 is due to the diprotonated adduct of the ligand [L + 2H]^2+^ (*m*/*z* 171.10), followed by the related monoprotonated species [L + H]^+^. (*m*/*z* 341.19). The peak at *m*/*z* 402.11 is ascribed to a 1:1 metal-to-ligand species [L + Cu − H]^+^. It is important to note that this complex species is already detected at pH 5, and it becomes the base peak from pH 6 to pH 8. The high stability of the Cu-GHK complex accounts for its formation at acidic pH. The attribution of the *m*/*z* 402.1 signal was confirmed by Zoom Scan analysis (Figure S10): the shape of the isotope pattern is typical of the presence of natural copper in the complex species.

The MS analysis of the Cu-GHK system was also performed for the Cu(II) complex with BioGHK (Figure S11). Although the pattern of the species in the MS spectra between the two systems looks very similar, there are some particular differences. First of all, the main complex species (*m*/*z* 321.61) of the Cu-BioGHK system is due to a double-charged ion ([L+Cu]^2+^), whereas the main species of the Cu(II)-GHK system is single-charged. Furthermore, the signal relating to the main complex species of copper with BioGHK (*m*/*z* 321.6) is the base peak already at pH 5.

In order to study the interaction between Cu(II) and the new synthesized derivative, Circular Dichroism (CD) was employed.

In the case of the Cu-GHK complex, the CD spectrum in the 260–750 nm region does not change appreciably between pH 5 and 6, while at pH 7 the absorption bands have a slight shift which does not influence their intensity (Figure S12A). These data, together with the UV–viss data (Figure S12B), confirm what has already been observed using ESI-MS, i.e., the main complex species does not change from pH 5 to 7. It was not possible to analyze solutions at higher pH because the system precipitates after pH 7, as confirmed by baseline rise in spectrophotometric measurement (Figure S12B, inset graph).

### 2.6. Effect of GHK and Derivative on Aβ Carbonylation

Oxidative stress is one of the main pathological features of neurodegenerative disorders (ND), such as AD. The production of carbonylated species can induce post-translation modifications on endogenous proteins, such as Aβ, thus triggering pathological processes.

In order to evaluate the production of carbonylated species of Aβ, carbonylation reactions were carried out using acrolein (ACR) as reactive carbonyl species since high levels of this exogenous aldehyde have been detected in the brain of AD patients [26]; moreover, the administration of ACR in vivo leads to an increase of the Aβ and phosphorylated Tau protein levels [27].

The amyloid peptide fragment (Aβ_1–16_) linked to a small PEG chain on the C-terminal (Figure S13) was used as a substrate of the ACR-induced carbonylation process. The solubility of this modified peptide and the stability toward the self-induced aggregation are higher than that reported for the most common form of the Aβ protein in vivo (Aβ_1–40_ or Aβ_1–42_). Moreover, Aβ_1–16_ contains most of the reaction sites of the Aβ protein that could be involved in the ACR-mediated carbonylation process (N-terminal amino group, His, Lys and Arg). All these aspects make Aβ_1–16_ a good model of the amyloid protein to investigate the carbonylation reactions.

ACR can react with the N-terminal amino group, the two imidazole nitrogen atoms of the three His residues and the amino group of lysine, meaning that Aβ_1–16_ can bind up to eight molecules of ACR, giving rise to the formation of both Michael adducts and Schiff bases and related rearrangements (Figure S14). All Michael adducts (M+56) are still reactive and unstable because of the presence of the aldehyde group. For this reason, we used NaBH_4_ to reduce the aldehyde group to a stable alcoholic one (M+58, Figure S15). All the Schiff bases are formed through a substitution reaction, with the consequent elimination of a water molecule (M+38, Figure S15).

**Figure 5:**
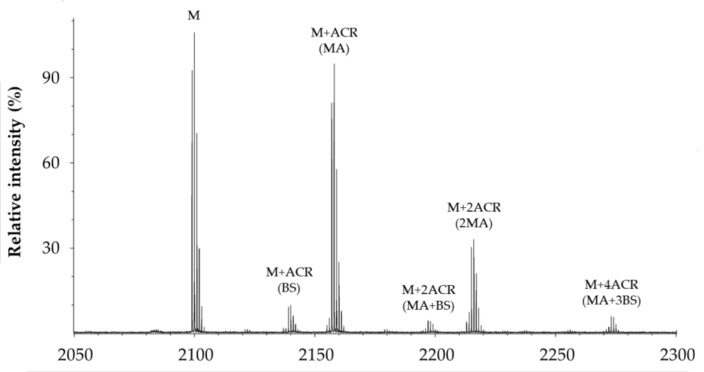
Representative MALDI spectrum of products formed by the reaction between PEG-linked Aβ_1–16_ and ACR.

Figure 5 shows a representative MALDI spectrum of the products formed by the reaction between the modified Aβ_1–16_ and ACR. In addition to the monoprotonated (M) species of the peptide (*m*/*z* 2099.955), the spectrum mainly contains the reduced form of mono-charged Michael adducts detected at *m*/*z* M+58. All the species formed by the reaction between the amyloid substrate and ACR, in the absence and the presence of GHK or BioGHK, and detected by MALDI-TOF mass spectrometry, are reported in Table 2.

All the species reported in Table 1 are mono-charged (z = 1) protonated adducts of the peptide substrate, modified or not with ACR. Adducts with Na^+^ and K^+^, although detected in the MALDI spectra, have a much lower intensity than that of those related to the protonated species. The main species revealed are Michael adducts (MA), Schiff bases (BS) and related cyclic rearrangements (MP). The adducts having the higher mass are due to the formation of Aβ_1–16_ fragments that bind up to six ACR molecules.

In order to better compare the results obtained, the intensity of all protein adducts containing the same number of ACR units and detected in the same spectrum were combined and compared as a function of the incubation time and the concentration of GHK (Figure S16).

The area graph related to the carbonylated species formed upon the reaction between Aβ and ACR (Figure S16A) clearly shows that the Aβ_1–16_ (M) content progressively decreases as the reaction proceeds. Conversely, the relative amount of the various carbonylated species increases as the reaction proceeds, especially for the species with the higher ACR content. In the presence of GHK (Figure S16B–D), the amount of Aβ_1–16_ still decreases over time, whereas the normalized areas related to the different carbonylated species increase as the reaction time increases (Figure S16A). However, the kinetic trend of Aβ_1–16_ and those of the related carbonylated species slightly changes as a function of the concentration of GHK. Indeed, Figure S17A shows the variation over time of non-carbonylated Aβ_1–16_. In the absence of GHK, the progressive decrease in Aβ is due to the formation of the ACR adducts. In the presence of GHK (Aβ/GHK molar ratio was 1:1, 1:2 and 1:3), the curves show the same trend, but the relative intensity of Aβ in the presence of GHK increases compared to that observed in the absence of the tripeptide. This effect, proportional to the concentration of GHK, can be reasonably ascribed to the formation of GHK-ACR adducts, which reduces the amount of ACR able to react with Aβ. As a matter of fact, taking into account the carbonylated species of Aβ having the higher molecular weight (i.e., those linking more than 3 ACR units) after 45 min reaction (Figure S17B), their relative amount decreases as the concentration of GHK increases. This result clearly confirms that GHK competes with Aβ for the reaction with ACR, thus reducing the formation of Aβ-ACR adducts.

**Figure 6:**
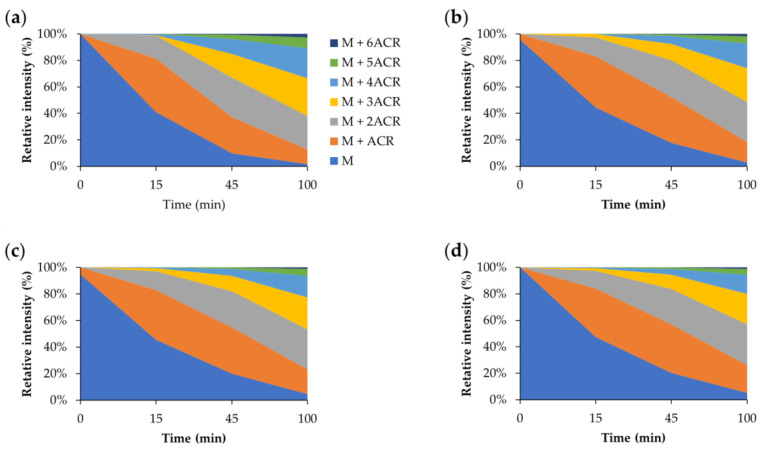
Relative intensity of all species detected by MALDI mass spectrometry following the reaction between Aβ and ACR (**a**) in the absence and in the presence of BioGHK, the Aβ:BioGHK molar ratio being (**b**) 1:1, (**c**) 1:2 and (**d**) 1:3.

**Figure 7:**
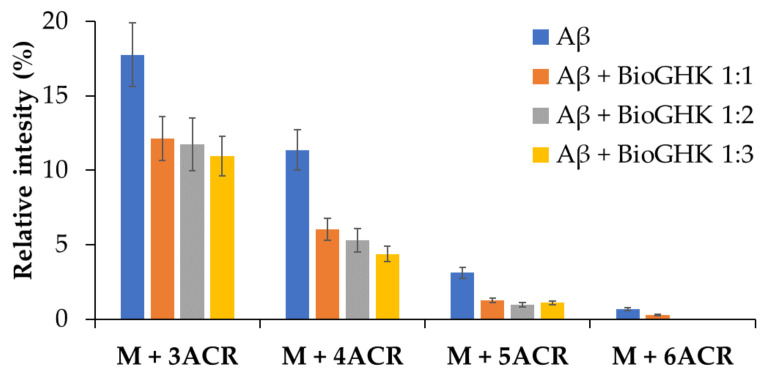
Relative intensity of the carbonylated species of Aβ at different concentrations of BioGHK, after 45 min reaction.

The same investigation was carried out using BioGHK. The amount variation of Aβ_1–16_ and that of related carbonylated species, both in the absence and in the presence of BioGHK (Figure 6), follows the same trend reported in the presence of GHK (Figure S16). Also in this case, comparing the decrease of non-carbonylated Aβ_1–16_ (Figure S18) and the increment of the carbonylated species with the higher ACR content (Figure 7) reveals that the treatment with BioGHK induces the same effects observed in the presence of GHK. However, BioGHK seems to reduce the formation of Aβ carbonylated species at concentration values higher than those of GHK; such a difference could be due to the biotin unit linked to the carboxyl of GHK, partially affecting the ability of the tripeptide to react with ACR.

### 2.7. Effect of GHK and Derivative on Ascorbate Oxidation

The pro-oxidant activity of ascorbate (ASC) is limited, in vivo, to conditions where iron and copper ions are aberrantly coordinated, thus able to catalyze a Fenton-type reaction (Figure S19) [28]. In AD, copper ions are found in high concentrations (0.4 mM) in amyloid plaques; in vitro, Cu(Aβ) can catalyze the production of ROS in the presence of ASC, and it has been proposed that these ROS may contribute to the oxidative stress observed in AD.

The copper-induced oxidation of ASC was evaluated by monitoring the trend of the ASC absorption peak at 265 nm. Figure S20A shows that the absorption of ASC does not greatly change in the absence of copper(II), indicating that ASC decomposes very slowly in the absence of redox-active metal ions. In the presence of copper ions, on the other hand, the absorption decreases are quite evident and proportional to the Cu^2+^ concentration, meaning that the metal ion catalyzes the ASC oxidation. The UV absorption spectrum at the end of the incubation time (Figure S20B) confirms the effect of the metal ion on the ASC oxidation process.

We evaluated the effect that GHK and BioGHK have on the Cu-Aβ complex-induced ASC oxidation. In the absence of GHK, the ASC oxidation quickly occurs, the ASC content almost disappearing after 30 min (Figure S21A,B). The oxidation kinetic trend is not significantly modified by the addition of GHK at low concentration values (2 µM). Higher contents of GHK (5 and 10 µM) significantly affect the Cu-Aβ-induced ASC oxidation because the oxidation reaction rate is clearly slowed down. Such an effect is reasonably due to the formation of the Cu-GHK complex. The highest concentration of GHK tested (30 μM) makes the slope of the curve almost zero. This means that the tripeptide, in excess with respect to Aβ, coordinates all the copper content; the GHK-Cu complex species do not allow the redox reaction involving ASC oxidation.

**Figure 8:**
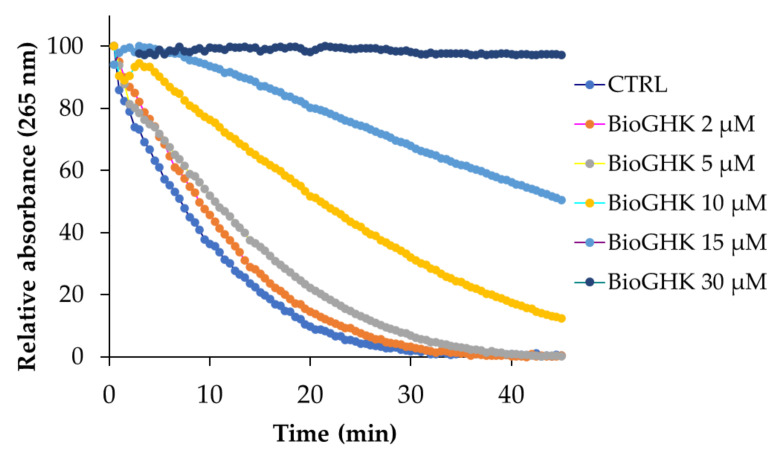
ASC oxidation kinetics induced by the Cu^2+^-Aβ complex in the absence and in the presence of BioGHK (0–30 µM).

The effect of BioGHK on the ASC oxidation catalyzed by the Cu-Aβ complex is shown in Figure 8 and Figure S22. Also in this case, the oxidation rate of ASC is modified by BioGHK in a dose-dependent manner.

Comparing the effects of GHK and BioGHK at the same concentration value (e.g., 10 µM), the inhibition activity of the former on ASC oxidation seems greater than the latter. Biotin unit could play a role in rationalizing such a difference. The C-terminal carboxylic group of GHK could be involved in the coordination of copper(II); the same group is linked to biotin in BioGHK and, therefore, cannot coordinate copper(II). Consequently, the stability constant of the main species of the Cu-BioGHK complex at physiological pH could be lower than that reported for the main species of the Cu-GHK system. The lower the copper(II)-binding ability, the lower the inhibitory activity towards the Cu-Aβ-mediated ASC oxidation.

### 2.8. Antiaggregant Activity towards Aβ

One of the main factors involved in the onset and/or progression of neurodegenerative disorders is the abnormal accumulation of intrinsically disordered proteins, whose natural folding state is somehow modified, and they become more prone to forming soluble and toxic oligomers first; the dimension of these initial aggregated species increases during the pathologic process, and fibrillary and insoluble structures form at the end of this pathway [29]. The negative interference on the aggregation process is one of the strategies to prevent the onset or attenuate the progression of these devastating disorders [30,31]. Therefore, BioGHK and the related parent compounds were tested for in vitro self-induced aggregation of Aβ_1–42_, the most toxic proteoform of Aβ, whose dyshomeostasis is involved in Alzheimer’s disease [32].

Kinetic measurements of the amyloid-type aggregation of Aβ_1–42_ were carried out in order to investigate the antiaggregant capabilities (if any) of BioGHK and the related parent compounds. The kinetic parameters of the aggregation process are the maximum fluorescence increment (*F_max_* − *F*_0_), proportional to the number of amyloid-type fibrils, and the lag time (*t_lag_*) that foregoes the aggregation *incipit*. Consequently, the higher t_lag_ and the lower *F_max_ − F*_0_ are, the better the antiaggregant activity.

As for Aβ alone, the sigmoid-shaped fluorescence increment (Figure S23) proves the amyloid-type aggregation mechanism. The lag phase and *F_max_* − *F*_0_ values (Table 3) are consistent with those previously reported [31]. When biotin is co-incubated with Aβ, *F_max_* − *F*_0_ slightly decreases, even though it is not significantly different from that reported by Aβ alone. On the contrary, GHK and its equimolar mixture with biotin (Bio + GHK) significantly reduce the *F_max_* − *F*_0_ value. BioGHK outperforms GHK and shows the greatest antiaggregant activity towards Aβ aggregation among all the tested compounds: the *F_max_* − *F*_0_ value is one-third of that reported by Aβ alone. Finally, the lag phase of Aβ does not significantly change when the protein is incubated with BioGHK, nor with the related parent compounds.

## 3. Materials and Methods

Unless specified, all reagents and solvents were purchased from Sigma-Aldrich (Milan, Italy).

The following reagents were used for the synthesis of BioGHK: biotinyl-hydrazide, GHK (Glycyl-histidyl-lysine, acetate salt, Bachem), BOC anhydride (t-butyloxy-pyrocarbonate anhydride), HOBT (N-hydroxybenzotriazole), DCC (N,N-dicyclohexylcarbodiimide), DMF (Dimethyl-formamide), TFA (trifluoroacetic acid). ACR was obtained from the corresponding ethyl diacetal after incubation in 0.1M of HCl for one hour at room temperature.

The synthesis reactions were monitored by Thin-Layer Chromatography (TLC) using plates with a UV-active silica gel layer at 254 nm (Macherey-Nagel, Pozzuoli (Naples), Italy); the eluent mixture was propanol, water, ammonia and ethyl acetate (5:3:2:1). A solution containing ninhydrin (1% *w*/*v*) in acetone was used for the amino group detection, whereas the Pauly’s test, a chromogenic reaction to detect imidazole and phenolic groups, consists of a solution of Fast Red B salt (Fluka, Brescia, Italy) 10% *w*/*v* in water.

The purification of the intermediate and final products was carried out by Flash Chromatography through the CombiFlash Rf apparatus (Teledyne Isco, Genova, Italy), using a 15.5 g Redisep Rf C18Aq Gold reverse-phase column and operating with water and methanol as the eluents.

^1^H NMR spectra (Varian INOVA, 500 MHz) were carried out in D_2_O or CD3OD; the structural characterization was studied using ESI Mass Spectrometry (LCQ Deca XP Max and Q-Orbitrap) (Thermo Scientific, Milan, Italy). In the latter case, the samples were dissolved in H2O/CH3OH 1:1, containing or not HCOOH 1%.

All the UV–vis and fluorimetric measurements of small volume samples (up to 300 µL) were performed in triplicate, using the Varioskan Flash Multimode spectrophotometric plate reader (Thermo Fisher Scientific, Milan, Iatly).

### 3.1. Synthesis of BioGHK

GHK (5.8 × 10^−4^ moles) reacts with BOC anhydride (2.35 × 10^−3^ moles) in methanol (30 mL) under constant stirring for 3 h [17]. The solution was dried, and the residue was purified by Flash chromatography in reverse phase, using a multistep gradient of water (Solvent A) and methanol (Solvent B), as well as UV detection to monitor the purification (210–230 nm). The flow rate was 30 mL/min, and 5 mL fractions were collected. The fractions containing the reaction product (GHK-di-BOC) were sampled and dried by lyophilization to obtain the intermediate product. Yield: 31%. ESI–MS: *m*/*z* 541.4 [M + H]^+^, *m*/*z* 563.4 [M + Na]^+^. ^1^H NMR (CD3OD) (Figure S24A): δ (ppm) 8.41 (s, 1H, e), 7.22 (s, 1H, e′), 4.71 (m, 1H, c), 4.25 (dd, 1H, f), 3.72 (dd, J1 21.3 Hz, J2 17.3 Hz, 2H, b), 3.20–3.39 (m, 2H, d), 3.02 (t, *J* 6.7 Hz, 2H, l), 1.95 (m, 2H, g), 1.72 (m, 2H, i), 1.32–1.57 (m, 20H, a,a′,h).

GHK-di-BOC (1.8 × 10^−4^ moles) was dissolved in anhydrous DMF (25 mL) together with HOBt (1.8 × 10^−4^ moles) and DCC (1.8 × 10^−4^ moles). After 30 min at room temperature and under constant stirring, biotinyl-hydrazide (2.7 × 10^−4^ moles) was added and the reaction was carried out for 48 h. The reaction mixture was then dried and TFA (5 mL) was added to the residue. After 2 h at room temperature, the solvent was removed and the residue was dissolved in water (30 mL). The precipitate was removed by filtration (0.45 µm), and the eluate was purified by Flash chromatography, using UV detection for monitoring the product species (210–230 nm). The elution is carried out by setting a multistep gradient of water (Solvent A) and methanol (Solvent B). The flow rate was 30 mL/min and 5 mL fractions were collected. The fractions containing the main product (BioGHK) were sampled, the solvent was dried and the product was lyophilized. Yield 9.58%. ESI–MS: *m*/*z* 581.4 [M + H]^+^, *m*/*z* 603.4 [M + Na]^+^, *m*/*z* 291.5 [M + 2H]^2+^. ^1^H NMR (D_2_O): δ (ppm) (Figure S24B): 8.51 (s, 1H, e), 7.21 (s, 1H, e′), 4.65 (m, 1H, c), 4.50 (dd, 1H, r′), 4.28–4.35 (m, 2H, r+f),3.74 (dd, 2H, b), 3.25 (m, 1H, q), 3.17 (dd, J1 15.3 Hz, J2 6.5 Hz, 1H, d), 3.09 (dd, J1 15.4 Hz, J2 7.4 Hz, 1H, d′), 2.897–2.93 (m, 3H, s,l), 2.67 (m, 1H, s′), 2.26 (t, *J* 7.2 Hz, 2H, m), 1.46–1.81 (m, 10H, g,i,o,p,n), 1.35 (m, 2H, h).

### 3.2. Interaction with Streptavidin

In order to calculate the number of binding sites of streptavidin (Sav), to a solution of Sav (5 µM, 200 µL), in phosphate buffer (10 mM, pH 7.4), a solution of HABA (2- (4-hydroxyphenylazo) benzoic acid, 2 mM) was added at regular intervals (7 min, 40 µL of total added volume), measuring the absorbance at 500 nm [24].

To determine the affinity of the new synthetic derivative with Sav, compared to that of biotin, a back-titration of the Sav-HABA complex is performed with the BioGHK derivative or with biotin. A solution of Sav (10 µM, 200 µL) saturated with HABA (50 µM), in phosphate buffer (10 mM, pH 7.4), was titrated with BioGHK (2 mM) or biotin (2 mM), the absorbance being recorded at 500 nm.

### 3.3. Copper(II) Complexes

The structural characterization of the new conjugate was carried out by multidimensional fragmentation (MS^2–4^) using the ESI LCQ Deca XP Max Mass Spectrometer (Thermo Scientific). BioGHK, GHK and their Cu(II) complexes (1:1) were dissolved in H_2_O/CH_3_OH 9:1 to a final concentration of 10 µM. pH (from 4.0 to 9.0) was adjusted by adding NaOH 1M.

Circular dichroism studies were performed using a J-810 spectropolarimeter (JASCO, Milan, Italy). The Cu-GHK and Cu-BioGHK 1:1 complexes were prepared using CuSO_4_. The concentration of the complexes was 2 × 10^−4^ M (210–280 nm) or/and 1 × 10^−3^ M (260–750 nm). The same solutions were used for the spectrophotometric measurements, using the V-670 spectrophotometer (JASCO, Milan, Italy).

### 3.4. Carbonylation of Aβ

In order to evaluate the effect of GHK and BioGHK on the Aβ carbonylation by ACR, Aβ_1–16_ (40 µM) was solubilized in MOPS buffer (75 mM, pH 7.4), together with ACR (2 mM) and GHK or BioGHK (0, 40, 80 and 120 µM). The reaction was carried out at 37 °C. The reaction, after 0, 15, 45 or 100 min, was inhibited by adding NaBH_4_ (0.2 M). The samples were diluted 1:9 with water and analyzed by MALDI-TOF mass spectrometry (TOF/TOF™ 5800, SCIEX, Milan, Italy) using a pulsed nitrogen laser (337 nm). The matrix was a saturated solution of α-cyano-4-hydroxycinnamic acid in H_2_O/CH_3_CN/TFA 50:50:0.3. The samples were diluted 1:1 with the matrix. The calibrant contains a mixture of proteins within the *m*/*z* range of 904–3000.

The acquisition of the spectra relating to the ACR/Aβ adducts was obtained by setting the range *m*/*z* at 1800–2500 and the focus mass at *m*/*z* 2100. The intensity of the laser beam was progressively increased until obtaining a constant ionic current and a low background noise.

### 3.5. Oxidation of Ascorbic Acid Catalyzed by Cu^2+^-Aβ

The oxidation of ASC catalyzed by the Cu^2+^-Aβ complex [33] was carried out by solubilizing ascorbic acid (200 µM), CuSO_4_ (10 µM) and Aβ_1–16_ (12 µM) in phosphate buffer (100 mM, pH 7.4). GHK and BioGHK were also added (0–30 μM). Samples were incubated in a 384-well microwell plate, at 25 °C up to 45 min, the absorbance being monitored at 265 nm every 30 s.

### 3.6. Aβ Aggregation Assay

In order to monitor the effect of the BioGHK and the related parent compounds on the amyloid-type aggregation of Aβ, we carried out an assay previously reported [34]. Briefly, all the compounds of interest were incubated with Aβ_42_ (20 µM) and ThT (60 µM) in MOPS buffer (50 mM, pH 7.4) at 37 °C. The collected fluorimetric data (excitation and emission wavelengths were 450 nm and 480 nm, respectively) of all the measurements, carried out in triplicate, were fitted to Equation (2).

(2) $$F(t)=\frac{F_{max}-F_{0}}{1+e^{-\frac{t-t_{½}}{k}}}$$

*F_max_* − *F*_0_ is the maximum fluorescence increment during the amyloid-type aggregation value. The lag phase (*t_lag_*), that is, the time period before the formation of amyloid species, can be calculated by using Equation (3).

(3) tlag=t½−2k

The parameters of each set of measurements were expressed as the mean ± SD.

## 4. Conclusions

The multifactorial origin of neurodegenerative disorders has prompted scientific research towards new molecules that have a natural origin and could exert many beneficial activities to address several biomolecular issues of these pathologies.

The new biotin–GHK conjugate (BioGHK) preserves the copper(II)-binding ability of the tripeptide. Therefore, both the Cu(II) complexes with GHK and BioGHK inhibit the ROS production catalyzed by the Cu(II)-Aβ complex system. Moreover, BioGHK shows the antiglycating activity of the tripeptide towards the formation of carbonylated species of amyloid-β.

All the mentioned properties of BioGHK are comparable or slightly lower than that reported by GHK. However, the biotin residue provides beneficial effects to the new system, because it makes BioGHK capable of interacting with streptavidin, a well-known approach for drug delivery. Finally, BioGHK has an inhibitory effect towards the amyloid-type aggregation of Aβ higher than that reported by GHK, biotin or a mixture of them. Therefore, the beneficial effects due to the biotin conjugation abundantly counteract the mild inhibition of the antioxidant/antiglycant properties with respect to the action of GHK.

All the results of our investigation suggest that the new biotinylated GHK could be used as a therapeutic agent to treat and/or prevent several adverse biochemical reactions that characterize neurodegenerative disorders, such as Alzheimer’s disease.

## Figures and Tables

**Table 1:** Detected and calculated *m*/*z* values of species in the MS/MS spectrum formed upon fragmentation of the protonated form of BioGHK (M).

Species	*m*/*z* Calc.	*m*/*z* Exp.
M	581.30	581.31
M–H_2_O	563.29	563.30
M–2H_2_O	545.28	545.27
y_3_	524.28	524.26
y_2_	387.22	387.23
b_3_	323.18	323.17
b_3_–H_2_O	305.17	305.16
b_3_–H_2_O–NH_3_	288.15	288.15
y_1_	259.12	259.11
b_2_	195.09	195.10
b_1_	58.03	58.03

**Table 2:** List of the species (reduced form) produced as a consequence of the reaction between PEG-linked Aβ_1–16_ (M) and ACR as Michael adducts (MA), Schiff bases (BS) and related cyclic rearrangements (MP).

Species	*m*/*z* Calc.	*m*/*z* Exp.	Δm
M	2099.064	2098.969	0.095
M + ACR (BS)	2137.010	2136.985	0.025
M + ACR (MA)	2157.106	2157.011	0.095
M + 2ACR (MP)	2177.169	2178.023	−0.854
M + 2ACR (MA + BS)	2195.121	2195.026	0.095
M + 2ACR (2MA)	2215.149	2215.053	0.096
M + 3ACR (MP + MA)	2235.207	2235.021	0.186
M + 3ACR (2MA + BS)	2253.166	2253.068	0.098
M + 4ACR (1MA + 3BS)	2271.179	2271.058	0.121
M + 3ACR (3MA)	2273.148	2273.094	0.054
M + 4ACR (2MA + 2BS)	2291.186	2291.048	0.138
M + 4ACR (MP + 2MA)	2293.252	2293.100	0.152
M + 4ACR (3MA + BS)	2311.207	2311.11	0.097
M + 5ACR (2MA + 3BS)	2329.140	2329.100	0.040
M + 4ACR	2331.229	2331.136	0.093
M + 5ACR (MP + 3MA)	2351.317	2351.140	0.177
M + 5ACR (4MA + BS)	2369.337	2369.152	0.185
M + 6ACR (3MA + 3BS)	2387.343	2387.142	0.201

**Table 3:** Kinetic parameters related to the aggregation of Aβ_1–42_ alone (CTRL) or co-incubated with BioGHK and the related parent compounds.

	CTRL	Bio	GHK	Bio + GHK	BioGHK
*F_max_ − F*_0_	23 ± 3	18 ± 4	15 ± 4	14 ± 2	6 ± 2
*t_lag_*	26 ± 6	25 ± 4	25 ± 5	23 ± 3	24 ± 7

## Data Availability

Data are contained within the article and Supplementary Materials.

## Supplementary Materials

The following supporting information can be downloaded at: https://www.mdpi.com/article/10.3390/molecules28186724/s1, Figure S1: Chromatograms related to purification of (A) GHK-diBOC and (B) BioGHK; Figure S2: ^1^H NMR spectrum of GHK-di-BOC (CDOD, 500 MHz); Figure S3: (A) COSY and (B) TOCSY spectra of BioGHK; Figure S4: ESI-MS spectrum of BioGHK; Figure S5: *Zoom Scan* of the signals relating to the species (A) [M + 2H]^2+^, (B) [M + H]^+^ and (C) [2M + H]^+^ (M = GHK); Figure S6: Multidimensional fragmentation of BioGHK, whose the selected mono-charged protonated adduct (A: *m/z* 581.3, C: *m/z* 323.2, E: *m/z* 305.8) were fragmented to produce different signals (B: MS2@581.3, D: MS3@323.2, F: MS4@ 305.8); Figure S7: ESI-MS theoretical fragmentation scheme of BioGHK; Figure S8: Spectrophotometric titration of Sav-HABA with BioGHK. The arrow shows the decrease trend of the absorption band centered at 500 nm; Figure S9: ESI-MS spectra of Cu(II)-GHK complex at various pH values (5–8); Figure S10: *Zoom scan* of the main complex species of the Cu-GHK system at pH 7; Figure S11: ESI-MS spectra of Cu(II)/BioGHK 1:1 at various pH values (5–8); Figure S12: (A) CD spectra of the Cu(II) complex (L = BioGHK), B) absorbance of the BioGHK/Cu(II) complex in the Vis region; Figure S13: Aβ_1–16_ amino acid sequence with a PEG residue; Figure S14: Main adducts between ACR and the histidine and lysine side chains; Figure S15; Reduction of the aldehyde group of the Michael adduct by NaBH_4_ (upper) and formation of the Schiff base (lower); Figure S16: Relative intensity of all species detected by MALDI mass spectrometry following the reaction between Aβ and ACR in the absence (A) and in the presence of GHK, the Aβ:GHK molar ratio being 1:1 (B), 1:2 (C) and 1:3 (D); Figure S17: (A) Time-dependent amount of non-carbonylated Aβ in the absence and in the presence of increasing concentrations of GHK, (B) Relative intensity of the carbonylated species of Aβ at increasing concentrations of GHK, after 45 min of reaction; Figure S18: Variation of non-carbonylated Aβ over time in the absence and in the presence of increasing concentrations of BioGHK; Figure S19: ROS formation determined by the Cu-Aβ complex, in the presence of ASC; Figure S20: (A) Variation of the absorption of ASC (200 µM) at 265 nm, in the absence and presence of Cu^2+^ (5 and 10 µM), (B) Absorption spectrum of ASC after incubation for 30 min in the absence and in the presence of Cu^2+^ (5 and 10 µM); Figure S21: (A) ASC oxidation kinetics induced by the Cu^2+^-Aβ complex in the absence and in the presence of GHK (0–30 µM), (B) Absorption spectra of ASC (200 µM) after incubation for 45 min, in the absence and in the presence of GHK (0–30 µM); Figure S22: Absorption spectra of ASC (200 µM) after incubation for 45 min, in the absence and in the presence of BioGHK (0–30 µM); Figure S23: Kinetic profiles of Aβ_1–42_ aggregation alone (Aβ, 20 µM), incubated with biotin (Bio), GHK, an equimolar mixture of Bio and GHK (Bio + GHK), and BioGHK, the Aβ/compound molar ratio being 1:1; Figure S24: Proton assignment by NMR of GHK-di-BOC (A) and BioGHK (B).

## Sample Availability

Samples are not available.
