# Supplementary material for: New Biotinylated GHK and Related Copper(II) Complex: Antioxidant and Antiglycant Properties In Vitro against Neurodegenerative Disorders
Source: Molecules. 2023 Sep 20;28(18):6724. doi: 10.3390/molecules28186724 (PMC10538196; doi:10.3390/molecules28186724)
Supplement: Supplementary file 1 [file molecules-28-06724-s001.zip › molecules-2564429-supplementary.pdf]

SUPPORTING INFORMATION

New biotinylated GHK and related copper(II) complex: antioxidant and antiglycant properties against neurodegenerative disorders

Rita Tosto[1], Graziella Vecchio[2] and Francesco Bellia[1]*

[1] *Institute of Crystallography, CNR, P. Gaifami 18, 95126 – Catania (Italy)*

[2] *Department of Chemical Sciences, University of Catania, A. Doria 6, 95125 – Catania (Italy)*

* corresponding author: francesco.bellia@cnr.it

A

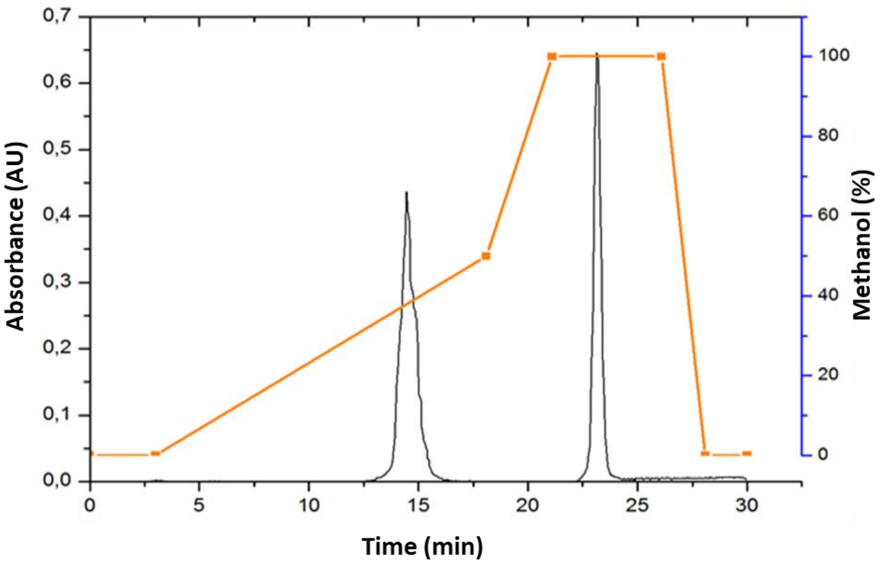

B

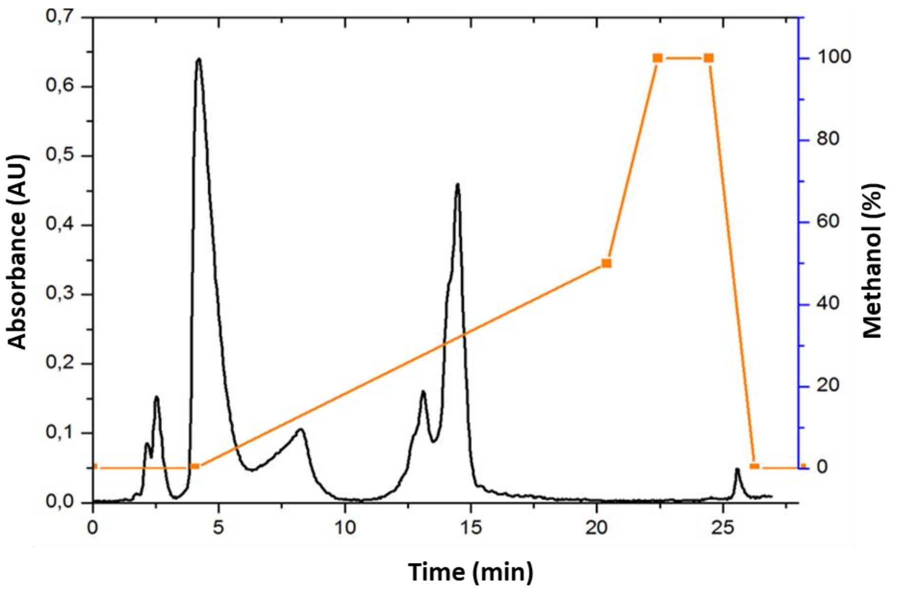

Figure S1. Chromatograms related to purification of (A) GHK-diBOC and (B) BioGHK

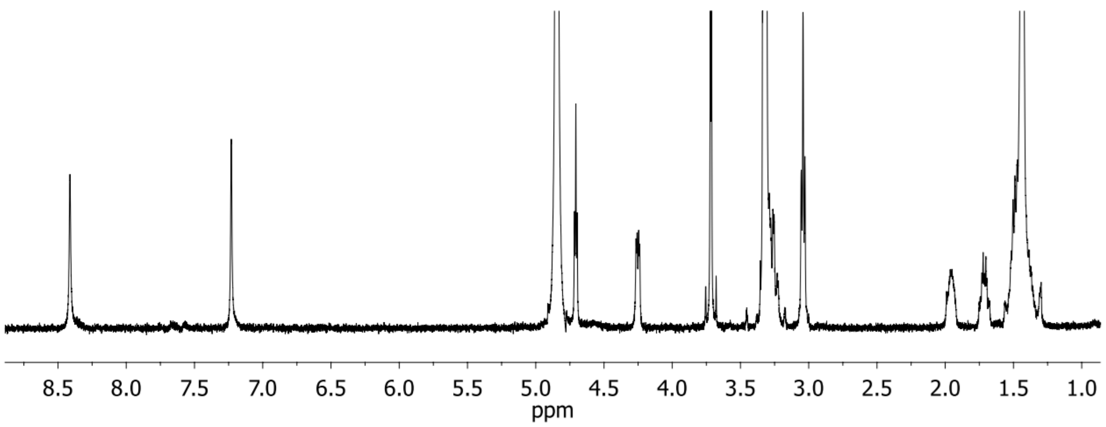

Figure S2. ^{1}H NMR spectrum of GHK-di-BOC (CDOD, 500 MHz).

A

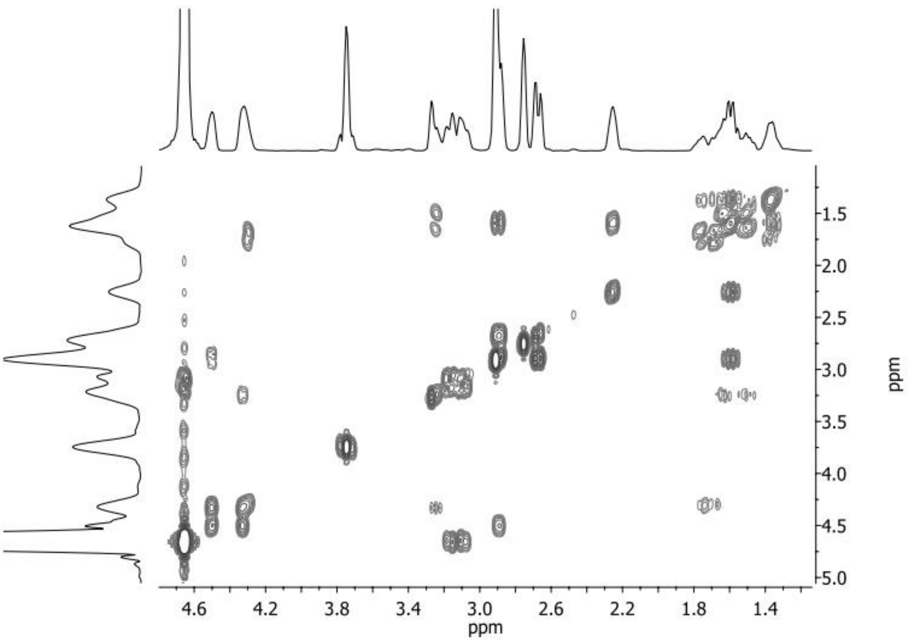

B

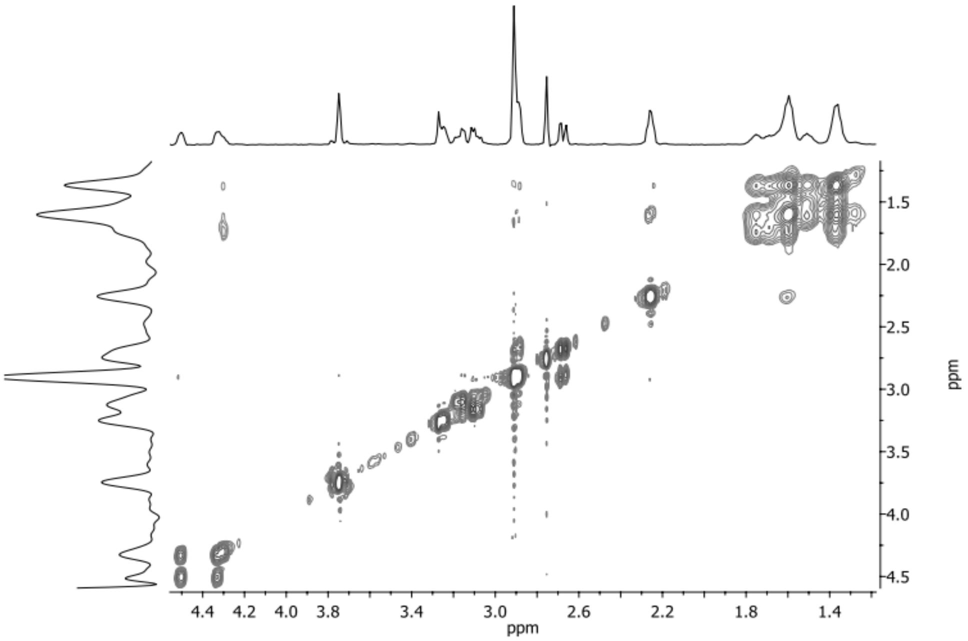

Figure S3. (A) COSY and (B) TOCSY spectrra of BioGHK.

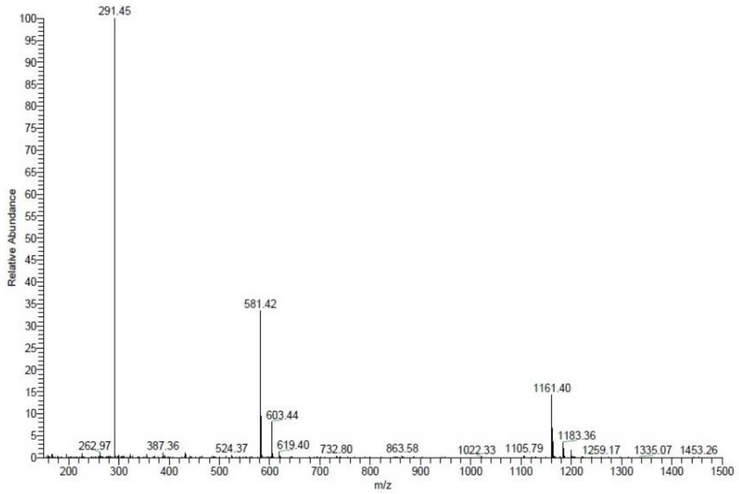

Figure S4. ESI-MS spectrum of BioGHK.

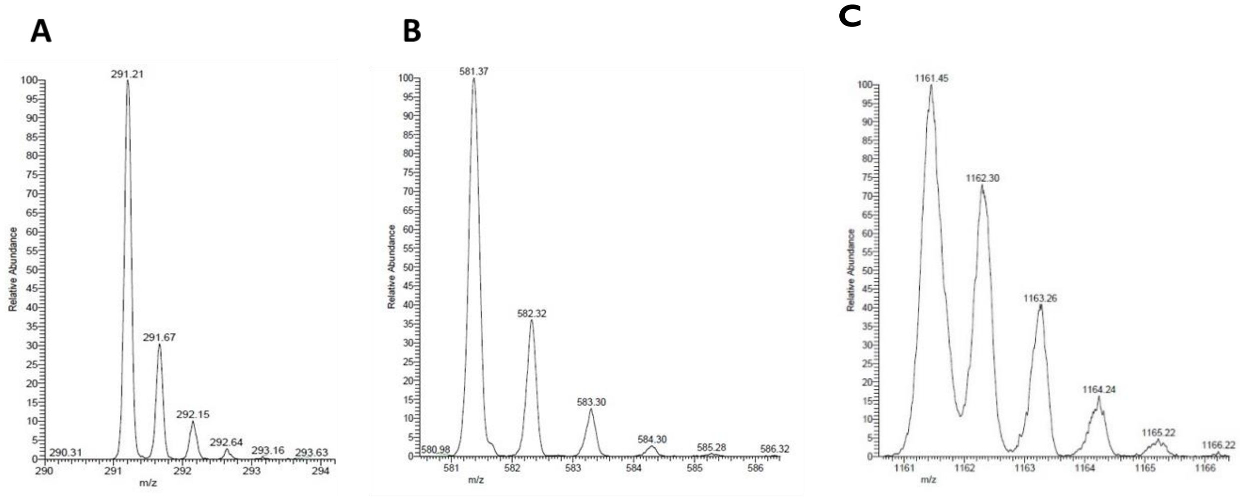

Figure S5. *Zoom Scan* of the signals relating to the species (A) $[M+2H]^{2+}$, (B) $[M+H]^{+}$ and (C) $[2M+H]^{+}$ (M = GHK).

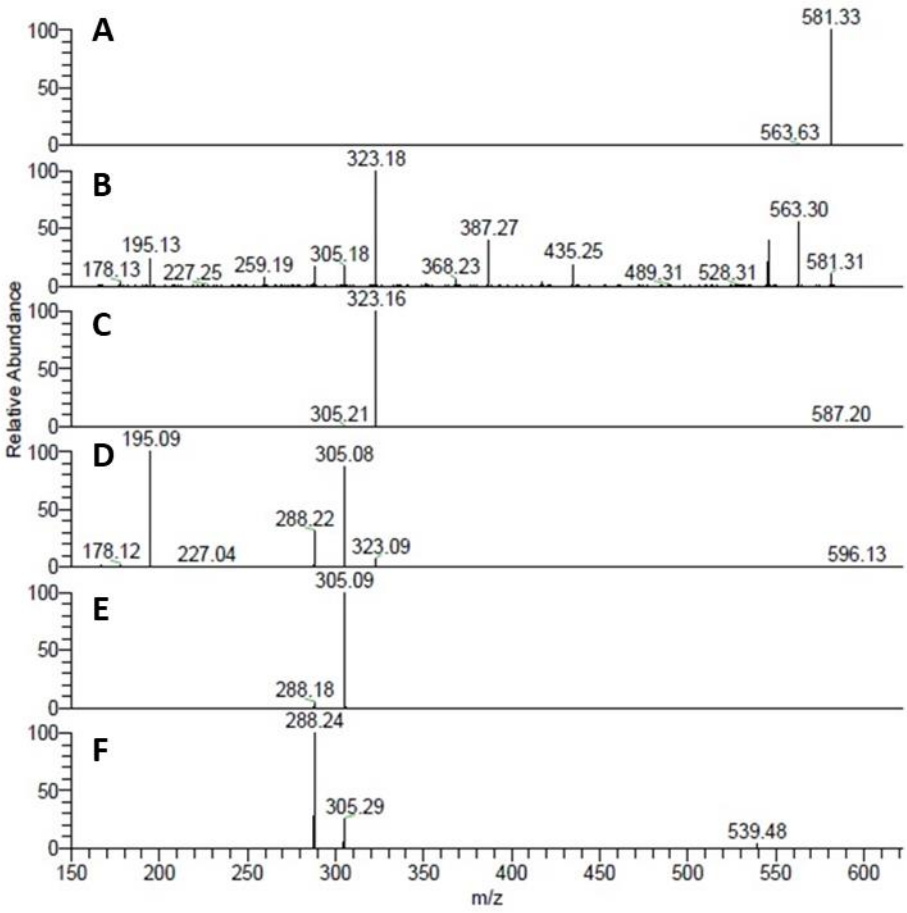

Figure S6. Multidimensional fragmentation of BioGHK, whose the selected mono-charged protonated adduct (A: *m/z* 581.3, C: *m/z* 323.2, E: *m/z* 305.8) were fragmented to produce different signals (B: MS2@581.3, D: MS3@323.2, F: MS4@ 305.8)

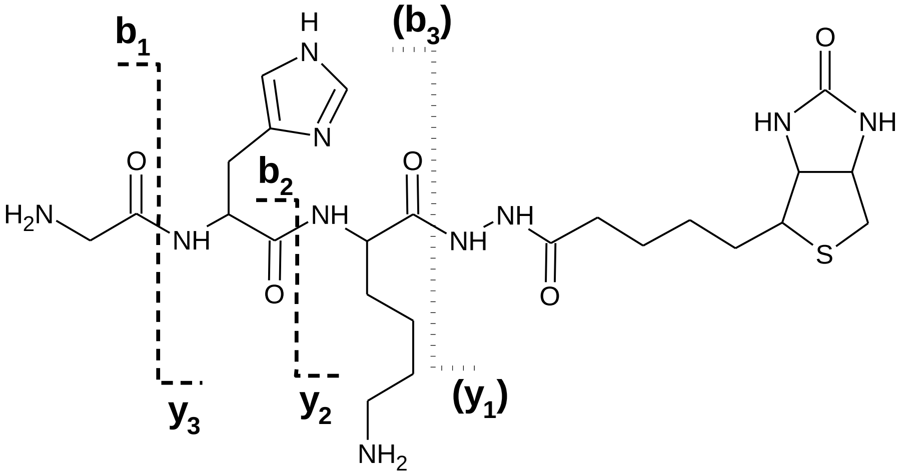

Figure S7. ESI-MS theoretical fragmentation scheme of BioGHK.

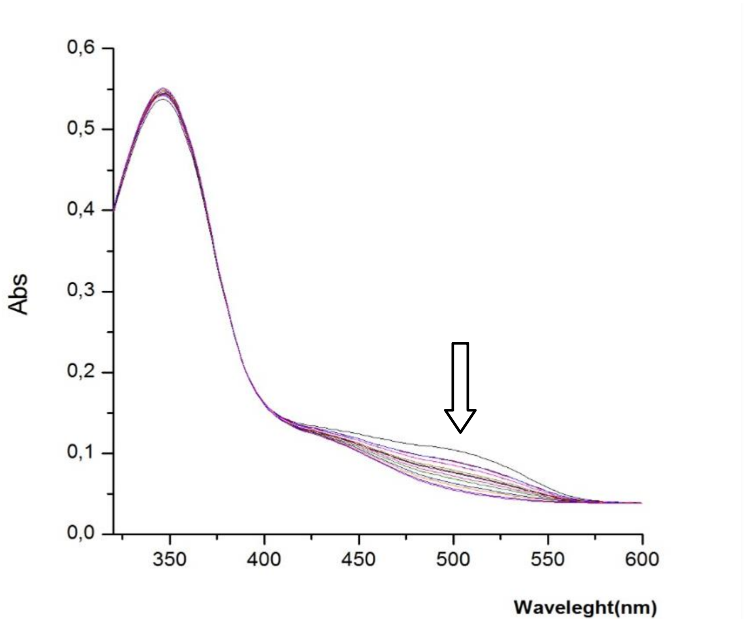

Figure S8. Spectrophotometric titration of Sav-HABA with BioGHK. The arrow shows the decrese trend of the absorption band centered at 500 nm.

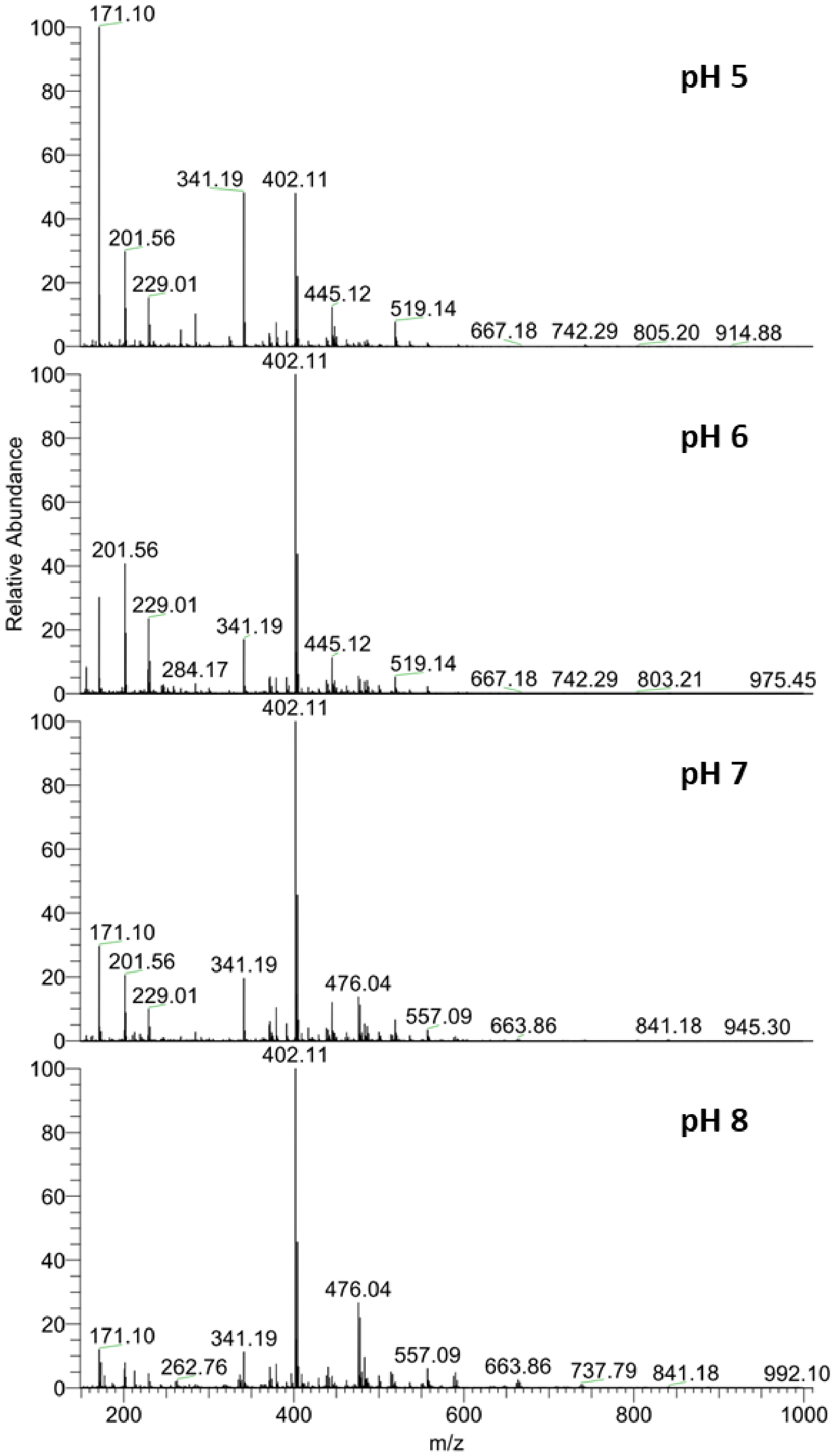

Figure S9. ESI-MS spectra of Cu(II)-GHK complex at various pH values (5-8).

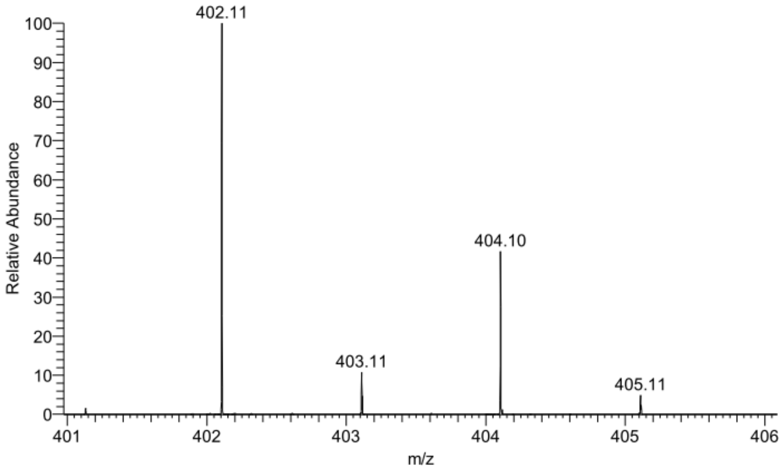

Figure S10. *Zoom scan* of the main complex species of the Cu-GHK system at pH 7.

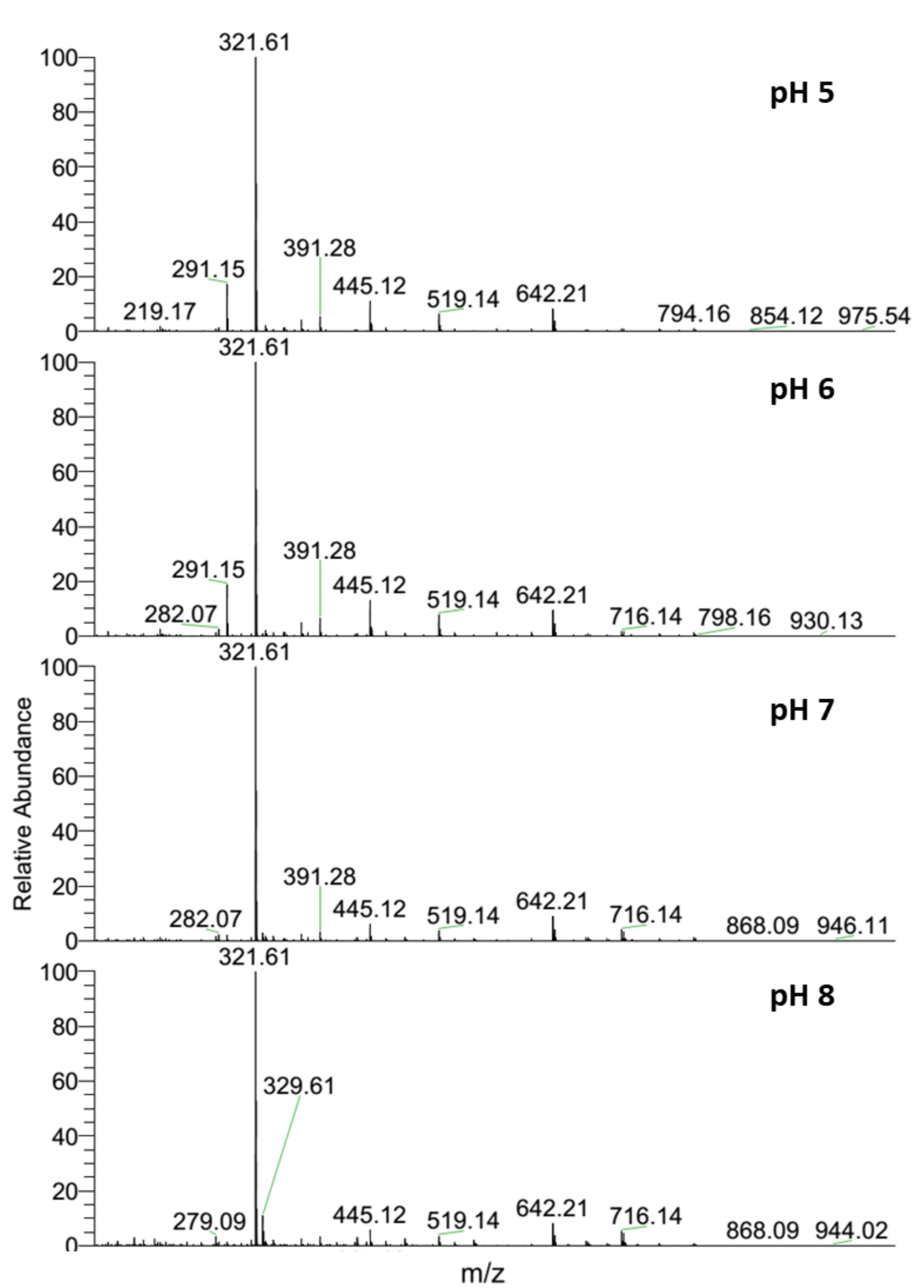

Figure S11. ESI-MS spectra of Cu(II):BioGHK 1:1 at various pH values (5-8).

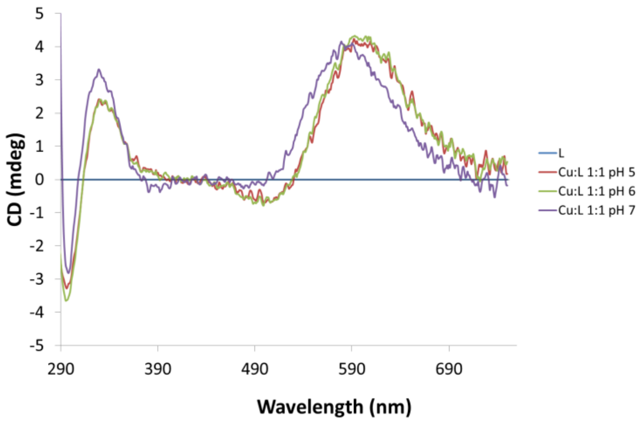

A

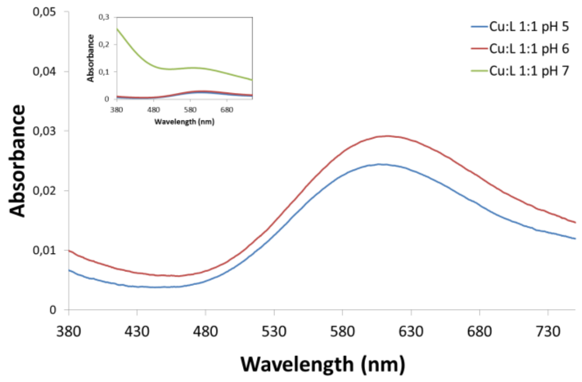

B

Figure S12. A) CD spectra of the Cu(II) complex (L = BioGHK); B) absorbance of the BioGHK/Cu(II) complex in the Vis region.

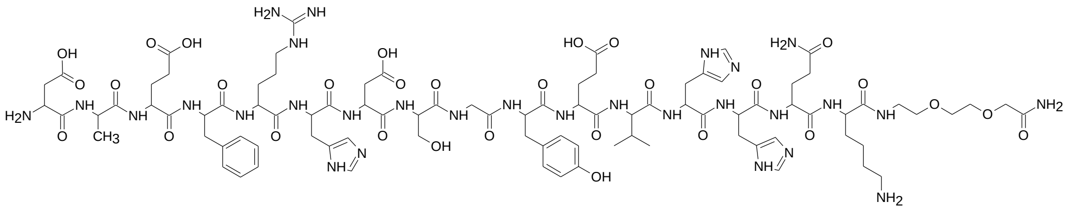

Figure S13. A$\beta_{1\text{-}16}$ amino acid sequence with a PEG residue.

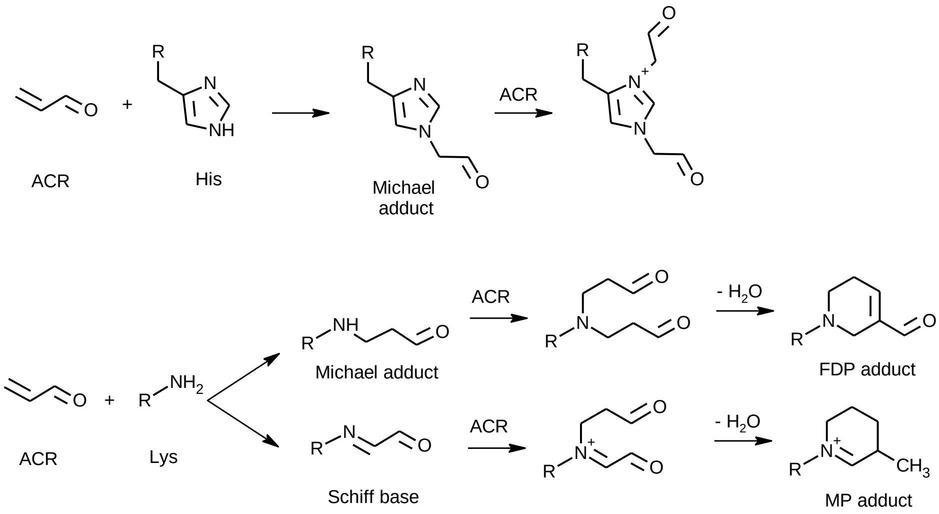

Figure S14. Main adducts between ACR and the histidine and lysine side-chains.

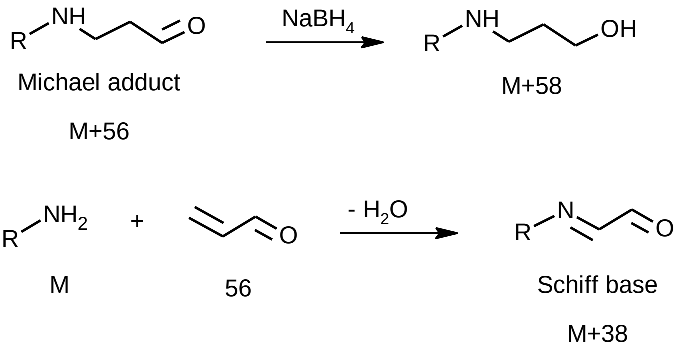

Figure S15. Reduction of the aldehyde group of the Michael adduct by $NaBH_4$ (upper) and formation of the Schiff base (lower).

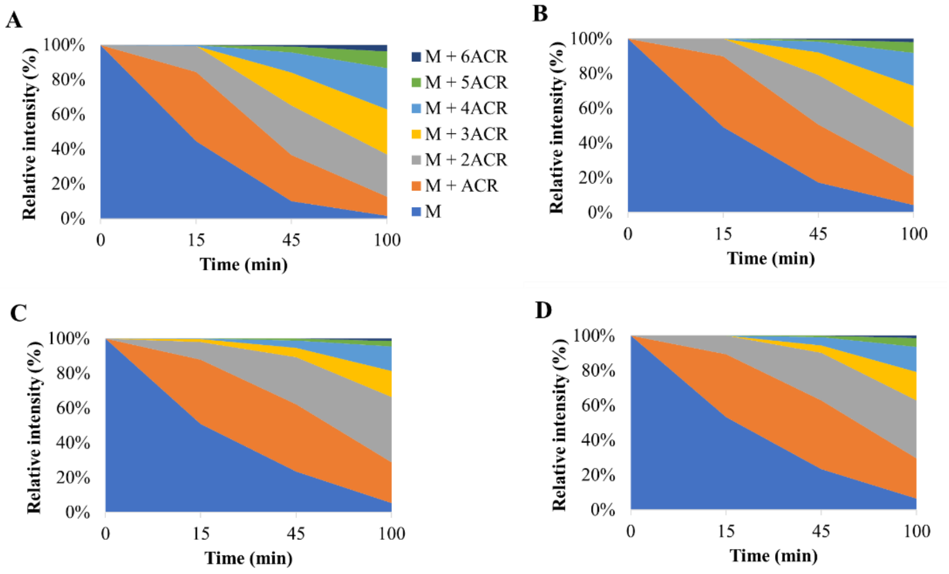

Figure S16. Relative intensity of all species detected by MALDI mass spectrometry following the reaction between Aβ and ACR in the absence (A) and in the presence of GHK, the Aβ:GHK molar ratio being 1:1 (B), 1:2 (C) and 1:3 (D)

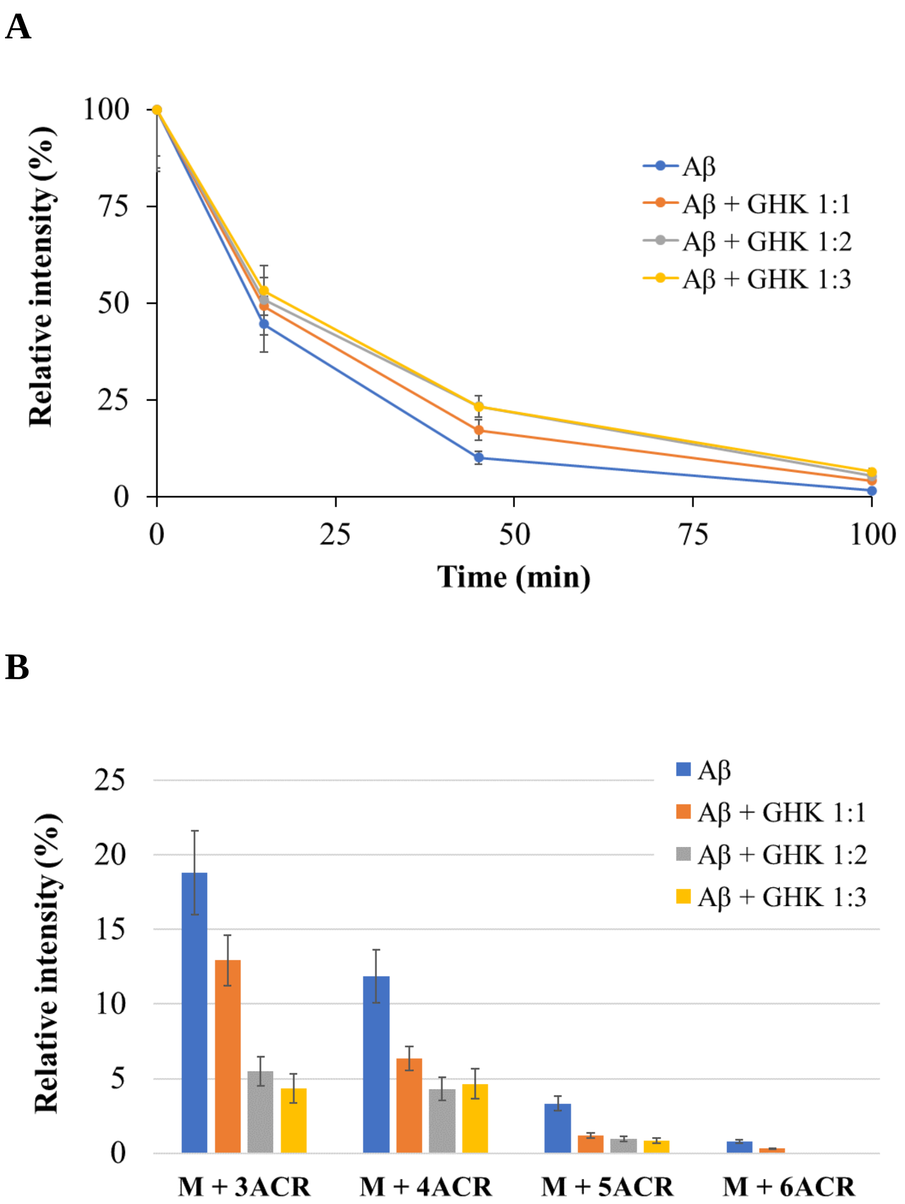

Figure S17. (A) Time-dependent amount of non-carbonylated Aβ in the absence and in the presence of increasing concentrations of GHK. (B) Relative intensity of the carbonylated species of Aβ at increasing concentrations of GHK, after 45 minutes of reaction.

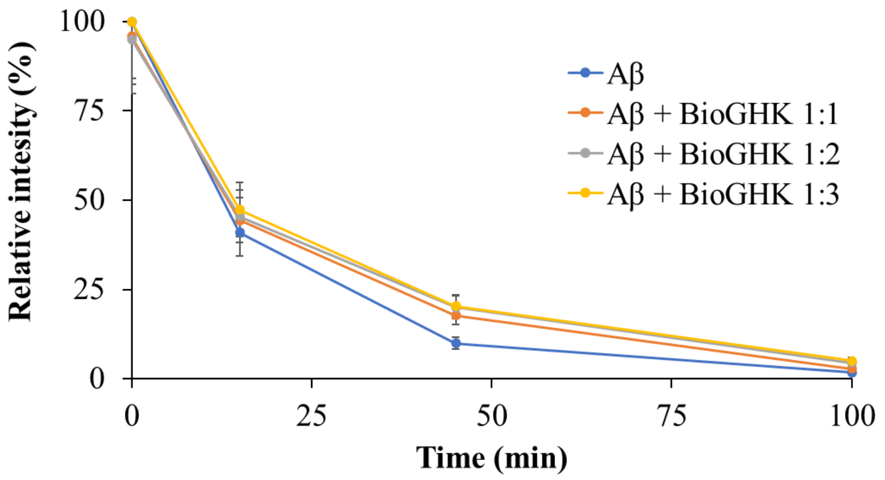

Figure S18: Variation of non-carbonylated Aβ over time in the absence and in the presence of increasing concentrations of BioGHK.

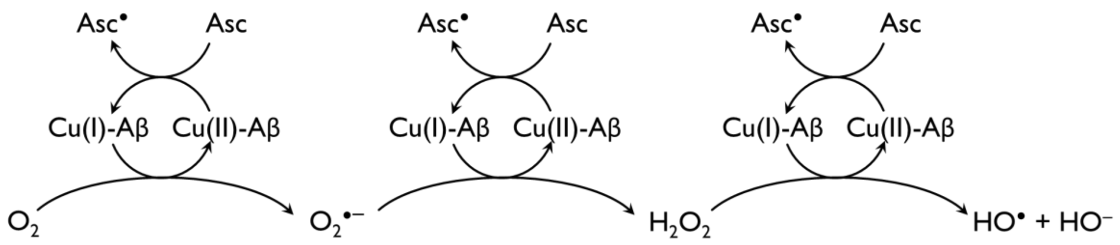

Figure S19. ROS formation determined by the Cu-Aβ complex, in the presence of ascorbate.

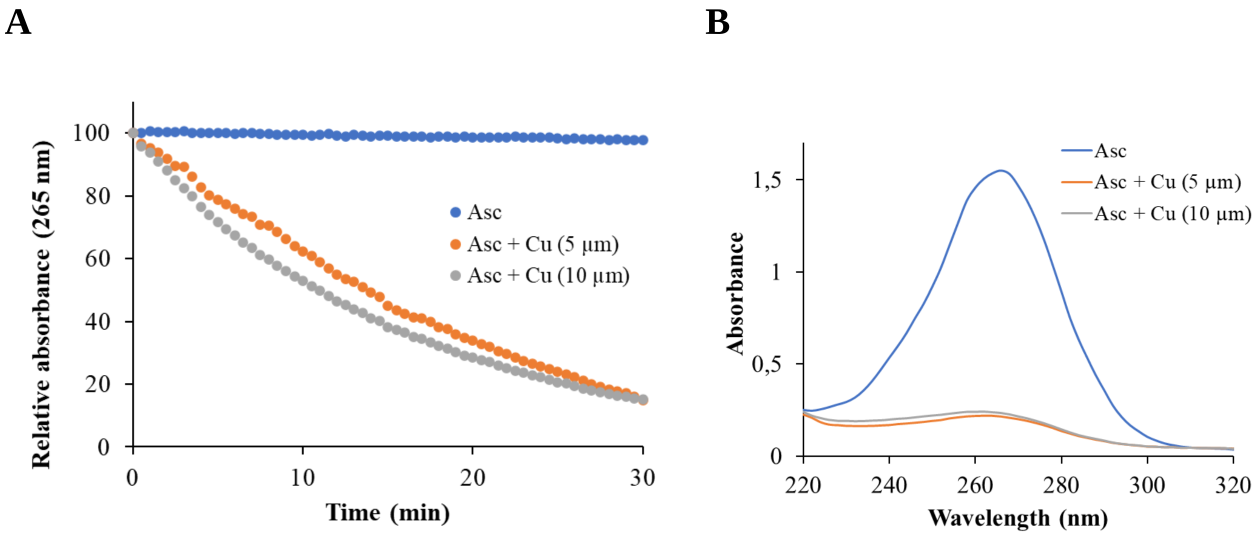

Figure S20. (A) Variation of the absorption of Asc (200 µM) at 265 nm, in the absence and presence of Cu^{2+} (5 and 10 µM). (B) Absorption spectrum of Asc after incubation for 30 min in the absence and in the presence of Cu^{2+} (5 and 10 µM).

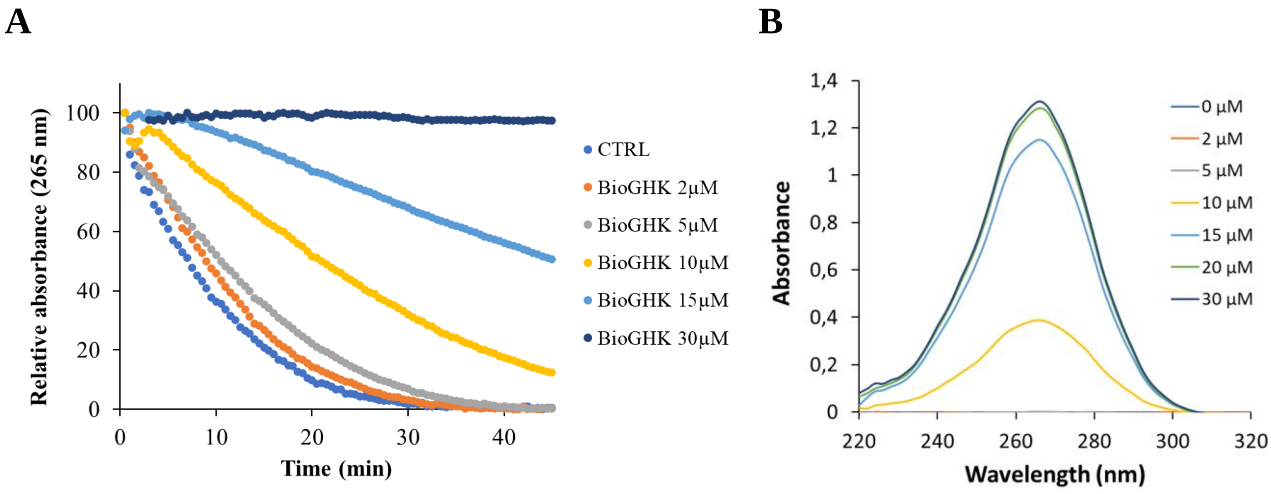

Figure S21. (A) Asc oxidation kinetics induced by the Cu^{2+}-Aβ complex in the absence and in the presence of GHK (0-30 µM). (B) Absorption spectra of ascorbate (200 µM) after incubation for 45 min, in the absence and in the presence of GHK (0-30 µM).

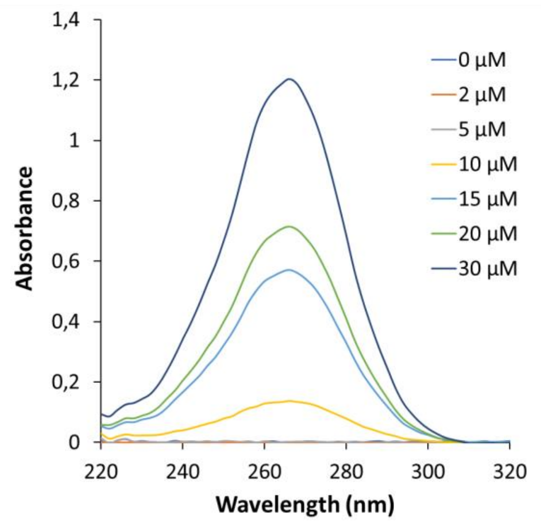

Figure S22. Absorption spectra of ascorbate (200 µM) after incubation for 45 min, in the absence and in the presence of BioGHK (0-30 µM).

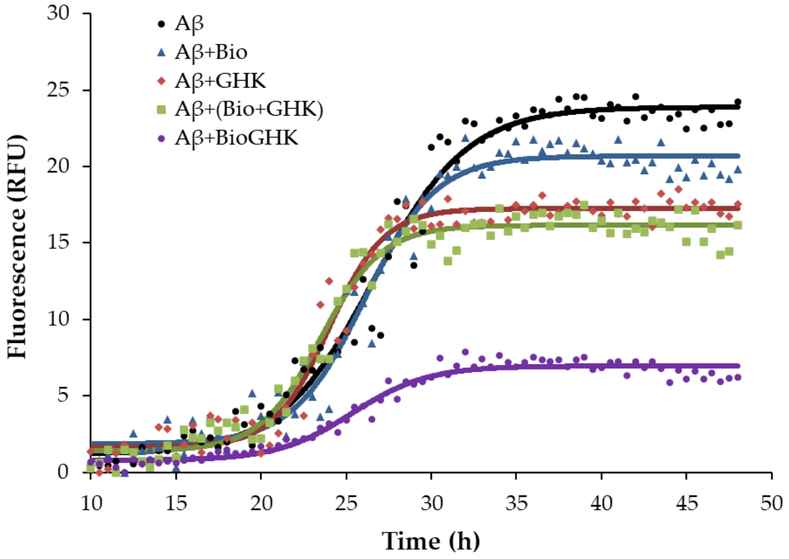

Figure S23. Kinetic profiles of Aβ_{1-42} aggregation alone (Aβ, 20 μM), incubated with biotin (Bio), GHK, an equimolar mixture of Bio and GHK (Bio+GHK), and BioGHK, the Aβ/compound molar ratio being 1:1.

A

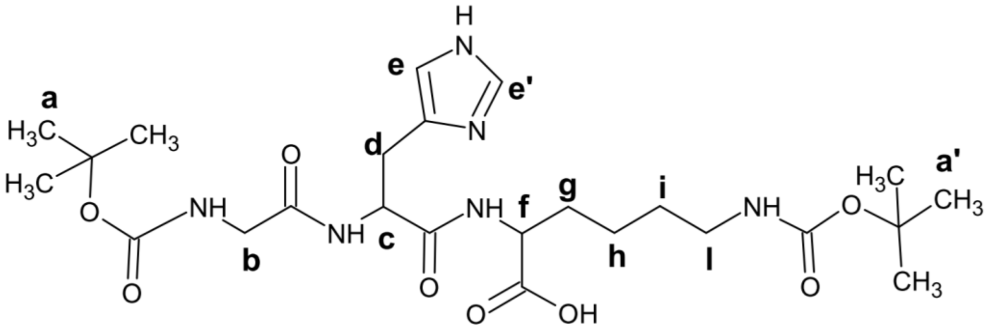

B

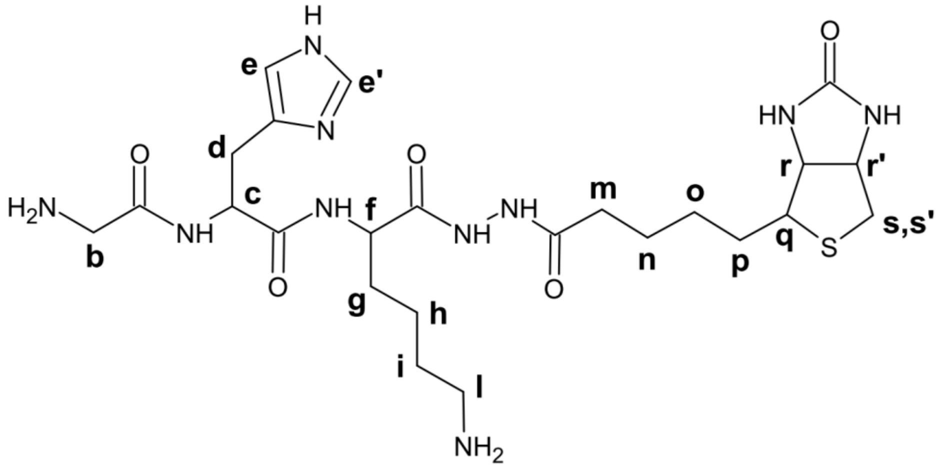

Figure S24. Proton assignment by NMR of GHK-di-BOC (A) and BioGHK (B).
